# Social network isolation and cohesion and their association with HIV clustering among Black and Latino sexual minority men: implications for public health strategy

**DOI:** 10.1038/s41598-026-45390-8

**Published:** 2026-04-24

**Authors:** Chi Fang, Ann M. Dennis, Dalton M. Craven, Annalea Greifinger, Justin Quimbo, Kham S. K. Piang, Britt Skaathun

**Affiliations:** 1 Department of Political Science, University of California, San Diego, La Jolla, CA USA; 2 Division of Infectious Diseases, University of North Carolina at Chapel Hill, Chapel Hill, NC USA; 3 North Carolina Department of Health and Human Services, Raleigh, NC USA; 4 Division of Infectious Diseases and Global Public Health, Department of Medicine, University of California, San Diego, La Jolla, CA USA

**Keywords:** Social network analysis, HIV/AIDS, Molecular epidemiology, Black/African–American, Hispanic/Latino, Diseases, Health care, Medical research, Risk factors

## Abstract

Evidence shows social networks shape HIV transmission by influencing partner access, behaviors, and health. Molecular cluster analysis offers insight into recent transmission, yet little is known about how network isolation or cohesion relate to clustering. This study examined social networks to identify factors associated with molecular HIV clustering. Data are from a cross-sectional study among Black and Latinx sexual minority men collected 2022–24 in Raleigh-Durham, NC. Participants nominated up to 20 friends and 20 sex partners. Other survey measures included age, gender, and socioeconomic status. Dyadic homophily was calculated using Jaccard similarity for race, STI status, drug use, and ethnicity. Network measures included the number of alters and a constraint score reflecting embeddedness. Molecular clusters were identified using pol sequences and nextHIV2. Ordinal logistic regression assessed associations with HIV status and cluster membership, adjusting for covariates. Among 100 participants (387 contacts), 60% were HIV-negative, 18% were people living with HIV (PWH) not in clusters, and 22% were PWH in clusters. Contact networks were sparse. Older age and more contacts were associated with cluster membership. Racial homophily increased odds of clustering, whereas larger networks, lower constraint, and greater STI negative status similarity reduced odds. Findings indicate that homophily and network structure are associated with HIV clustering and may inform HIV prevention strategies.

## Introduction

The study of HIV transmission has paid substantial attention to individual behaviors, such as condom use, substance use, and the number of sexual partners^1–3^. However, social and physical environments have emerged as a critical factor in the understanding of HIV transmission in more recent studies^4–7^.

Previous research has shown that social networks (defined here as relationships between individuals) are critical to the transmission of HIV. For example, lacking network structures that foster active propagation can limit HIV transmission even when individuals who participate in behaviors that increase HIV transmission are present^8^. Existing research also demonstrates that social networks can influence HIV transmission, either via the introduction of individuals with norms that put them at risk for HIV, or via communities that facilitate home-based HIV testing and provide information about improving HIV or general health outcomes^6,9,10^. Examining social networks has also advanced HIV prevention and intervention efforts by helping researchers more effectively recruit individuals with HIV to improve care, and identify potential HIV transmission pathways^11^.

Social networks entail not only connection but also disconnection. Some individuals are more isolated than others in social networks^12^. These disconnected individuals provide a valuable opportunity to analyze the transmission of information. Current research has demonstrated that information sometimes goes beyond tightly connected groups through weakly connected individuals who can bridge distant communities^13^. People living with and at elevated risk for HIV tend to be stigmatized and discriminated against in many social settings. Such isolation may further lead to a lower likelihood of being diagnosed and treated. For example, persons living with HIV who are named by fewer people tend to face a higher risks for poor HIV intervention. Specifically, older men and older adults of color are more likely to experience social isolation, a situation improved only if a person has a confident or receives instrumental support^14,15^. Unfortunately, it remains unclear how individuals form isolated groups and why some groups face more risks than others. While current studies pointed out the importance of age in understanding isolated people living with HIV (PWH), their variations in social network structures, relationships, and other behaviors remained underexplored^16,17^.

Moreover, molecular HIV epidemiology, through pairwise genetic distance or phylogenetic analyses of HIV sequences, can be used for genetic cluster detection. Highly similar viruses between individual persons indicate a shared common ancestor; these “clusters” on a population level can capture the underlying transmission network^18^. Molecular HIV epidemiology provides a framework to monitory the dynamics of HIV transmission and is increasingly used to guide interventions^19^.

Building on the recognition that social networks encompass both connection and isolation, and that these dynamics can shape HIV transmission, we sought to analyze ego networks (a person’s immediate connections) to identify behavioral, relational, and social network properties associated with the probability of molecular HIV clustering. Here, an ego refers to the participant who is at the center of sub-networks being analyzed. In these networks, an alter refers to individuals named by a participant (ego). In examining both connected and disconnected positions within social networks, we aim to uncover patterns that are not readily visible through traditional epidemiologic approaches. The results, composed of bivariate analyses and ordinal logistic models, unveil the determinants of HIV cluster memberships. This outcome informs more nuanced and targeted public health interventions that account for the diversity of social experiences among people with HIV.

## Data and methods

### Population and data collection

Data comes from a cross-sectional study conducted among sexual and gender minority (SGM) of color in Raleigh-Durham, North Carolina, which asked participants about their sexual networks as well as their experiences of discrimination. This quantitative study was designed to investigate networks with a potentially higher probability of HIV transmission-acquisition. Data was collected between June 16, 2022, and March 1, 2024. Eligibility criteria included age 18 years or older, Black/African American race or Latinx/Hispanic ethnicity (race and ethnicity were defined using the National Center for Education Statistics definitions)^20^, assigned male sex at birth, able to provide consent in English, and report a history of sex with another person assigned male sex at birth. In addition, people living with HIV must have met clinical eligibility criteria, which included those 1) newly diagnosed with HIV (prior 12 months), 2) HIV viral load > 200 copies/mL in the past 12 months, or 3) no viral load reported in the past 12 months and referred for bridge counseling services. In-person recruitment occurred at clinics (51%), from social media (29%), and from other locations (20%), such as passive referrals from the health department, community-based organizations, and local social venues. Further details are described elsewhere^21^.

The study was approved by the Institutional Research Boards at the University of North Carolina at Chapel Hill and University of California at San Diego. All participants provided informed consent, and research was performed in accordance with the Declaration of Helsinki.

Following informed consent, participants completed a self-administered, web-based survey. The survey included a name generator, which asked them to name at most 20 friends and 20 sex partners they are close to who they had seen in the past 30 days, who are at least 18 years old, who lived in Raleigh-Durham region, and who they talked with about their sex life. Participants were asked to provide a first name and the initial of a last name or a nickname. Then, we asked them to describe five nominees for each type of relationship. We excluded participants from the bivariate and ordinal logistic analyses who did not name any alters, resulting in the exclusion of 10 egos.

### Individual-level measures

Individual-level measures include age, gender, and socioeconomic status (SES). While age is a continuous variable, gender is a categorical variable composed of seven categories: (1) Male, (2) Female, (3) Trans-male, (4) Trans-female, (5) Do not identify as female, male, or transgender, (6) Non-binary/genderqueer/gender fluid/agender, and (7) Another identity not listed. Given the distribution, we recoded the variable as a binary variable: (1) Male and (2) Transgender and Gender Diverse (TGD).

Then, we derived an ordinal variable for SES. This variable sums up the recoded level of education and financial status. There were seven levels of education: (1) Some high school or less, (2) High school grad, (3) Some college, (4) Associate degree, (5) Bachelor’s degree, (6) Graduate degree, and (7) Prefer not to answer. Those with a college degree and above were grouped as higher education, and anyone with less than a college degree was categorized as lower education to derive a binary variable.

We also asked participants about the frequency of not having enough money in the household for rent, food, or utilities in the past 6 months. Financial status was coded in three levels: (1) Never, (2) Sometimes, and (3) Often. Then, we reduced the original scale into a binary indicator in which individuals will be coded 1 if one ever experienced financial difficulties. Accordingly, we obtained an ordinal variable of three levels, defined as the SES score: (0) Experienced financial difficulties and received lower education, (1) Experienced financial difficulties or received lower education, (2) Never experienced financial difficulties and received higher education.

### Dyadic-level measures

We calculated the Jaccard similarity between an ego and an alter based on four different attributes: race, drug use, and ethnicity. In our data, we coded 1 if an individual self-identified as a specific race, used a specific drug in the last 12 months, and self-identified as Latino/Hispanic ethnicity. This information was also provided by the ego participant about each network nominee. Using these binary indicators, we calculated the similarity index for every dyad (ego-friend or ego-sex partner). This was achieved by dividing the intersection of an ego’s and an alter’s attributes by the union of the same set of attributes. These estimates allow us to identify homophily effects that describe if individuals with similar characteristics cluster as a group. Here, each similarity score has a maximum of 1 and a minimum of 0. A higher number indicates higher similarity, and a lower number suggests more differences.

We also created a binary variable, concordant STI negative status, to indicate whether a dyad shares similar self-reported sexually transmitted infections (STIs). In particular, our questionnaire asked if an ego has been diagnosed for certain STIs and if an ego knows an alter’s STI history. This variable is coded as 1 if neither an ego nor an alter have been diagnosed with any STIs and0 if either side have been diagnosed with STIs.

### Social and sexual network-level measures

We asked individuals to evaluate whether their friends knew each other. This information allows us to construct a series of friendship networks, despite no such counterpart for sex partners. We derive two different social network variables. The first, the number of nominees measures the number of friends and sex partners an individual nominates. This variable has a maximum of 36 and a minimum of 0. Most individuals, however, named fewer than 10 individuals in total.

Subsequently, the constraint score captures the extent to which an individual is constrained by social networks. In theory, the constraint score reflects the extent to which an ego connects different cohesive groups. A higher constraint score indicates less opportunities to connect to different groups and reflects that an ego is surrounded by a cohesive network. In contrast, a lower constraint score reflects more opportunities to connect to different groups and more sparse networks^22^. Here, this is done by calculating the extent to which an ego is connected to the rest of the alters^8^. This measure is calculated by using the igraph package in R. If an individual’s friends are mutually connected, the ego will be heavily constrained because there are fewer opportunities for the individuals to control the flow of information or social capital. Otherwise, there will be fewer restrictions on an individual so that they can easily control the flow. A higher constraint score indicates more constraints and less flexibility for an individual.

### Reconstruction of the HIV molecular transmission network

For participants with HIV, we analyzed membership in HIV genetic clusters using North Carolina Department of Public Health (NC-DPH) surveillance data. Partial HIV pol sequences (as part of routine drug resistance screening) were used to construct the molecular network, with samples available since October 2010^23^. An automated cluster analysis pipeline (nextHIV2) is used to detect and monitor genetic clusters prospectively; the pipeline is routinely updated with sequence, demographic, and laboratory data, as reported to NC surveillance^24^. Deposited sequences are codon aligned against a reference sequence (HXB2) using the program bealign (http://github.com/veg/bioext), assuming an HIV-BETWEEN-F amino acid substitution matrix. Aligned sequences covering partial pol region (1212 base pairs; HXB2 position 2253-3464) are used for clustering analysis. Clusters of related sequences are generated by comparing the pairwise genetic distances with TN-93 nucleotide substitution model^25^ with averaging of ambiguities, as implemented in the tn93 program (http://github.com/veg/tn93). We selected pairs with < 1.5% distance; all connected pairs between individuals were then linked to form clusters of interconnected nodes. This genetic distance threshold has been validated for identifying partners with direct or indirect epidemiological links^12,26^ and is used for molecular HIV surveillance in U.S. public health^6,27^. Our study followed an ethical framework informed by community-engaged and qualitative research conducted by our team in North Carolina, which explored community perspectives and concerns about MHE^28,29^. All analyses use de-identified surveillance data and nextHIV2 within secure, restricted environments, with strict safeguards for privacy and confidentiality.

### Estimation models

The outcome of interest is an individual’s HIV status and HIV cluster membership, defined by three levels. The first level indicates a negative status of HIV. The second level represents those who are living with HIV, but not in HIV clusters. The third level is people living with HIV who are in HIV clusters.

Because the dependent variable is ordinal, we use ordinal logistic models and different specifications to estimate the effects of various variables. We ran seven different model specifications to focus on separate factors (Table 3). From the first to fourth models, we focus on the dyadic effects to highlight how relational attributes affect a participant’s HIV Status and Cluster memberships. The fifth to sixth models focus on a participant’s network attributes to examine the effect of social environments. Specifically, we emphasize the role of friend and sex partner nomination and the constraint score. While the former is a social behavior, the latter reflects the social structure of participants’ networks. Finally, we include all coefficients in the seventh model to consider how individuals are affected by their relationship and social environment simultaneously. These incremental steps help us establish more robust interpretations with statistical support.

All models include control variables: age, gender, and SES. These are key characteristics highlighted by earlier studies on HIV transmission and isolated groups^16,17^. All analyses were conducted using R software.

## Results

### Study population

In our sample, we surveyed 100 individuals. Thus, we obtained information on 487 individuals: 100 surveyed, 247 nominated friends, and 140 nominated sex partners. Ego-level findings, including personal attributes, dyadic attributes, and network attributes, are shown in Table 1. Among our participants, 60% (n = 60) were HIV negative, 18% (n = 18) were people living with HIV but not belonging in any HIV clusters, and the rest 22% (n = 22) were LWH belonging to HIV clusters. In total, 88% (n = 88) of participants were male and 12% (n = 12) were TGD.

**Table 1:** Descriptive characteristics of study population.

Categorical variable	N	Category	Count	Proportion [95% CI]
HIV Cluster	100	HIV neg	60	60.0% [50%, 70%]
		PWH/No cluster	18	18.0% [11%, 27%]
		PWH/In cluster	22	22.0% [15%, 32%]
Gender	98	Male	88	90.0% [82%, 95%]
		TGD	10	10.0% [5%, 18%]
Race	100	White	13	13.0% [6.6%, 19.7%]
		American Indian	2	2.0% [0.2%, 6.5%]
		Alaskan Native	0	0.0% [0.0%, 3.4%]
		Asian	1	1.0% [0.0%, 5.1%]
		Native Hawaiian	0	0.0% [0.0%, 3.4%]
		Pacific Islander	1	1.0% [0.0%, 5.1%]
		Black/African American	82	82.0% [66.7%, 83.6%]
		Other	9	9.0% [3.9%, 15.2%]
Ethnicity^a^	100	Hispanic	21	21.0% [14%, 31%]
		Non-Hispanic	75	75.0% [67%, 84%]
STI, ego	100	Syphilis	38	38.0% [28.5%, 48.3%]
		Gonorrhea	44	44.0% [34.1%, 54.3%]
		Chlamydia	33	33.0% [23.9%, 43.1%]
		Herpes	6	31.0% [26.4%, 35.9%]
		HPV	3	6.0% [2.2%, 12.6%]
		None	34	34.0% [24.8%, 44.2%]
		Other	4	4.0% [1.1%, 9.9%]
STI, alter	387	Syphilis	31	8.0% [5.5%, 11.2%]
		Gonorrhea	40	10.3% [7.5%, 13.8%]
		Chlamydia	42	10.9% [7.9%, 14.4%]
		None	120	31.0% [26.4%, 35.9%]
Concordant STI Neg. Status	387	Yes	69	17.8% [14.1%, 22.0%]
		No	318	82.2% [78.0%, 85.9%]

Continuous variable	N	Median	IQR
Age	97	32	26.00–38.00
SES	99	4.00	3.00–5.00
N. of Nominees	100	4.00	2.00–7.25
Constraint Score	100	0.50	0.25–0.77
Ego Burden	100	1.00	0.00–2.00

Dyadic variable	N (alters)	Median	IQR
Race similarity	387	1	0.00–1.00
STI similarity	387	0	0.00–0.00
Drug use similarity	247	0	0.00–0.50
Ethnicity similarity	387	0	0.00–0.00

15 non-Black/African American respondents are Hispanic/Latino and 2 non-Black/African American respondents did not identify as Hispanic/Latino.

N, number of participants; CI, confidence interval; IQR, interquartile range; TGD, Transgender and Gender Diverse; SES, Socioeconomic Status. “Ego” refers to the primary study participant, while “Alter” refers to members of the ego’s social network. All similarity index are Jaccard similarity.

^a^73 participants identified themselves as black and non-Hispanic. 16 participants identified themselves as non-black and Hispanic. Among the latter, 8 considered themselves white and Hispanic. Full table can be found in the appendix.

Most of our participants were Black/African American (82%, n = 82). The other groups are Hispanic White^1^ (13%, n = 13), other (9%, n = 9), American Indian (2%, n = 2), Asian (1%, n = 1), and Pacific Islander (1%, n = 1). Among these 100 individuals, the median age was 32, and approximately 75% of the sample was under 40 years old. The average SES score is 0.64, indicating that most of our sample experienced financial difficulties and received lower education. (10 (n = 53), 1 (n = 29), 2 (n = 17).)

The average number of nominees, friends, and sex partners combined for each participant is 5.46. Most individuals nominated fewer than 5 people, with a minimum of 0 and a maximum of 36. The average constraint score was 0.5325, with the max being 1.13 and the minimum being 0.14. The nominees listed are consistent with current literature that indicates most people list their closest contact first^30^. This also corresponds to our questionnaire that asked specifically about friends or sex partners who they talked with about their sex life.

**Fig. 1:**
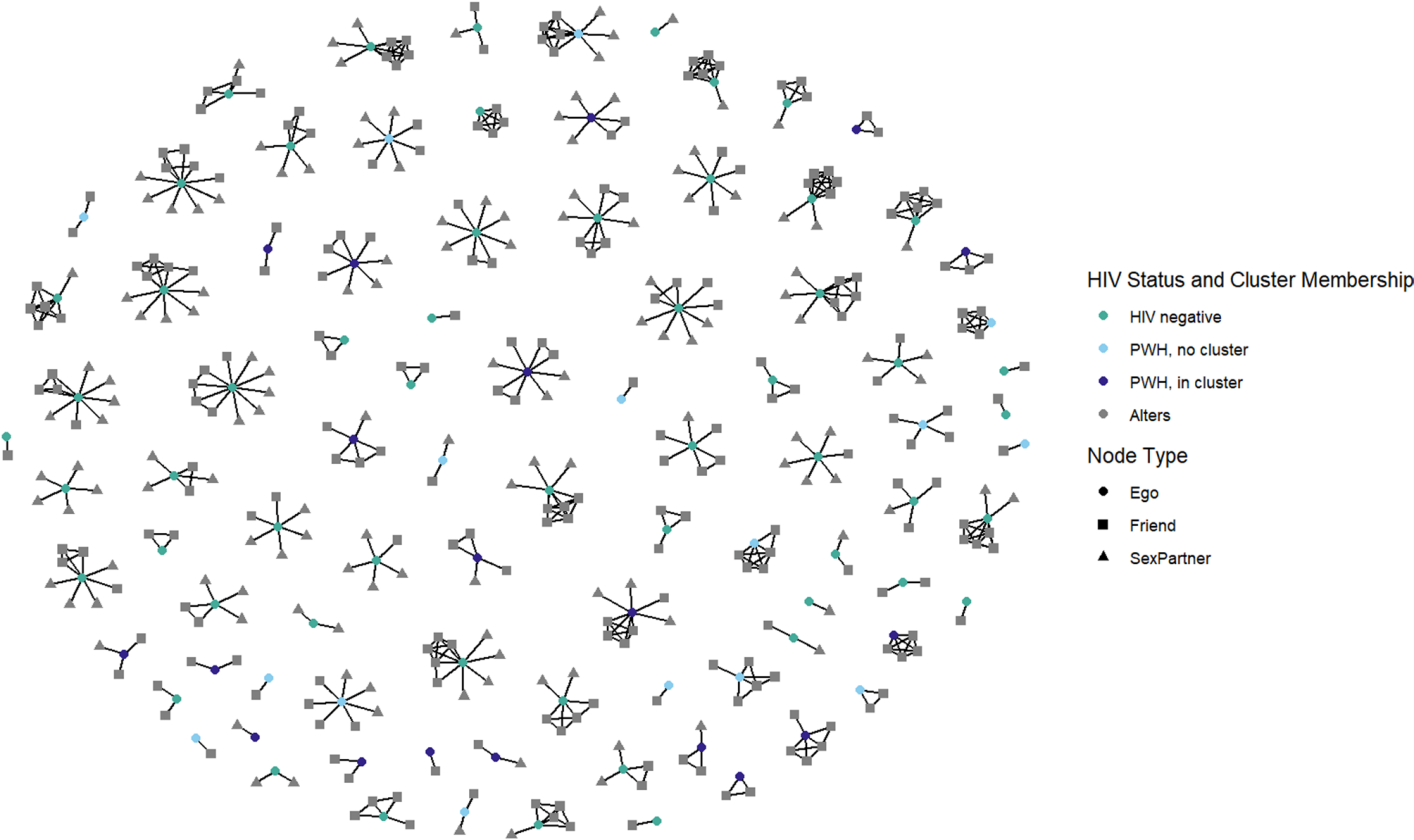
Ego networks. This figure shows 100 ego networks derived from our sample, with information on the egos’ HIV cluster membership and their connections. First, round nodes are egos, square nodes are friends, and triangle nodes are sex partners. Second, egos marked with blue nodes are HIV negative, yellow nodes are PWH not living in HIV clusters, and red nodes are PWH living in HIV clusters. The network structures are derived by using whether a respondent reports alters age as a proxy of their existence. We then asked whether friends know each other.

The network through which these indicators were derived is visualized in Fig. 1, which contains 100 ego-level networks, incorporating both friends and sex partners. Most of the network in this sample is star-shaped, suggesting the absence of connections among friends. However, there are more connections between friends than between sex partners. This could reflect a tendency to nominate primary, non-overlapping supports from their social networks. This is, however, a general limitation of name generators^31–33^.

### Bivariate analyses of cluster membership

Table 2 is a bivariate analysis of different variables’ associations with HIV status and cluster membership. It demonstrates statistical differences between ego demographic characteristics, dyadic similarities, and social network attributes by HIV status and cluster membership (HIV negative vs. living with HIV (LWH)/not in cluster vs. LWH/in cluster). First, age differed across groups (*p* = 0.04, SMD ∼ 0.72). Participants who were LWH but not in HIV clusters had the highest mean age (41.75, SD = 10.82), followed by those who were HIV negative (32.44, SD = 10.86), and then those who were PWH and in HIV clusters (31.06, SD = 7.30). Gender did not differ significantly across groups (*p* = 0.19, SMD ∼ 0.47). The proportions of males were similar across groups.

**Table 2:** Bivariate analysis participant characteristics by HIV status and cluster membership.

	HIV negative	LWH, no cluster	LWH, in cluster	*p*	SMD
N	55	16	19		
Age	32.44	41.75	31.06	0.04	0.723
	(10.86)	(10.82)	(7.30)		
Gender (%)				0.118	0.470
Male	48 (87.3)	15 (93.8)	18 (94.7)		
TGD	7 (12.7)	0 (0.0)	1 (5.3)		
SES	4.24	3.81	4.05	0.519	0.216
	(1.34)	(1.22)	(1.43)		
Race similarity	0.58	0.84	0.70	0.038	0.486
	(0.37)	(0.34)	(0.37)		
Concordant STI neg. status	0.29	0.05	0.11	0.022	0.519
	(0.41)	(0.19)	(0.27)		
Drug use similarity	0.23	0.32	0.34	0.452	0.196
	(0.32)	(0.39)	(0.36)		
Ethnicity similarity	0.06	0.03	0.03	0.600	0.159
	(0.13)	(0.12)	(0.11)		
N. of nominees	7.35	3.56	3.95	0.011	0.543
	(6.56)	(2.28)	(2.32)		
Constraint	0.48	0.64	0.58	0.170	0.330
	(0.31)	(0.34)	(0.31)		

Categorical variables use the Chi-square test, and continuous variables use the one-way test.

N, number of participants; CI, confidence interval; IQR, interquartile range; SMD, Standard Mean Difference; TGD, Transgender and Gender Diverse; SES, Socioeconomic Status. “Ego” refers to the primary study participant, while “Alter” refers to members of the ego’s social network. All similarity indices are Jaccard similarity.

Values are means; standard deviations in parentheses.

We removed 10 respondents who did not name any alters.

There were no significant differences across groups in terms of SES (*p* = 0.83). While not significant, participants who were HIV-negative had a higher SES score on average (1.19, SD = 0.59). Participants in the PWH/no-cluster group had a lower SES score on average (1.12, SD = 0.34), and those in the PWH/in-cluster group had the lowest SES score (1.11, SD = 0.57).

Some of the demographic and behavioral similarity indicators, as well as the social network attributes, were also similar across groups. However, there were significant differences in terms of the number of nominees (*p* = 0.01, SMD = 0.54), race similarity (*p* = 0.04, SMD = 0.49), and concordant health (*p* = 0.02, SMD = 0.52). Participants in the HIV-negative group nominated more friends and sex partners on average (7.35, SD = 6.56). Conversely, participants in the other groups only nominated 3.56 people (SD = 2.28) and 3.95 people (SD = 2.32), respectively. Next, participants in the LWH (0.84, SD = 0.34) and LWH and being in an HIV clusters (0.70, SD = 0.37) have a higher race similarity score than in the HIV-negative group (0.58, SD = 0.37). Finally, on average participants in the HIV-negative group have more alters with concordant STI status (0.29, SD = 0.41) than those LWH (0.05, SD = 0.19) not in HIV clusters or LWH and being in an HIV cluster (0.11, SD = 0.27).

### Estimation models

We ran multiple different model specifications to evaluate separate factors that may influence HIV status and cluster membership. Because we are interested in the formation of HIV clusters, we included both dyadic and network attributes while controlling individual characteristics. Table 3 presents multivariate analyses using dyadic data, including both ego-friend dyads and ego-sex partner dyads. The first four models focus on the dyadic effects. We can observe from Model 1 that concordant STI negative status plays an important role in an individual’s likelihood of LWH and being in HIV clusters. Compared to dyads in which either side has STIs, concordant STI status is associated with an 81% decrease in the odds of LWH or LWH and being in an HIV cluster (95% CI 0.08–0.47). The second model shows that race similarity is important to the formation of clusters. A one point increase in race similarity is associated with a 151% increase in the odds of LWH or LWH in an HIV cluster (95% CI 1.52–4.16). Other characteristics show no clear effect on cluster membership.

**Table 3:** Order logit estimation (Dyadic odds ratio): factors associated with HIV status and cluster membership.

	Model 1	Model 2	Model 3	Model 4	Model 5	Model 6	Model 7
Concordant STI Neg status	0.19 [0.08, 0.47] *p* = 0.0003						0.19 [0.06, 0.56] *p* = 0.003
Dyadic race similarity		2.51 [1.52, 4.16] *p* = 0.0003					1.09 [0.55, 2.17] *p* = 0.8073
Dyadic drug use similarity			1.83 [0.9, 3.72] *p* = 0.0942				1.56 [0.72, 3.39] *p* = 0.2618
Dyadic ethnicity similarity				0.4 [0.13, 1.23] *p* = 0.11			0.55 [0.12, 2.42] *p* = 0.4286
Ego’s N. of Nominees					0.77 [0.72, 0.83] *p* < 0.0001		0.68 [0.59, 0.78] *p* < 0.0001
Ego’s Constraint score among friend networks						3.52 [1.49, 8.34] *p* = 0.0044	0.1 [0.02, 0.45] *p* = 0.003
Age	1.02 [1, 1.04] *p* = 0.0954	1.02 [1, 1.05] *p* = 0.0367	1.04 [1.01, 1.07] *p* = 0.0069	1.03 [1.01, 1.05] *p* = 0.0129	1.02 [1, 1.04] *p* = 0.1059	1.03 [1, 1.05] *p* = 0.0222	1.01 [0.98, 1.04] *p* = 0.3984
Gender (TGD)	0.34 [0.14, 0.82] *p* = 0.0163	0.36 [0.15, 0.9] *p* = 0.0296	0.22 [0.07, 0.7] *p* = 0.0102	0.35 [0.14, 0.87] *p* = 0.0243	0.72 [0.28, 1.83] *p* = 0.4934	0.37 [0.15, 0.91] *p* = 0.0313	0.44 [0.13, 1.48] *p* = 0.1879
SES	0.93 [0.78, 1.11] *p* = 0.4337	0.87 [0.73, 1.04] *p* = 0.1378	0.88 [0.72, 1.09] *p* = 0.2454	0.93 [0.78, 1.1] *p* = 0.408	0.97 [0.81, 1.16] *p* = 0.7024	0.98 [0.83, 1.16] *p* = 0.8132	0.83 [0.65, 1.07] *p* = 0.1496
AIC	608.05	612.71	425.96	623.37	555.23	618.30	370.74
BIC	631.59	636.26	446.79	646.91	578.77	641.84	408.94
Log likelihood	− 298.02	− 300.36	− 206.98	− 305.68	− 271.61	− 303.15	− 174.37
Deviance	596.05	600.71	413.96	611.37	543.23	606.30	348.74
Num. obs	374	374	238	374	374	374	238

Odds ratios reported; 95% confidence interval standard in brackets.

The fifth and sixth models focus on a participant’s network structural attributes (the role of friend or sex partner nomination and the constraint score) on HIV status and cluster membership. Nominating one additional friend or sex partner was associated with a 23% decrease in the odds of LWH or LWH and being in an HIV cluster (95% CI 0.65–0.81). While a one-unit increase in the constraint score increased the odds of LWH or LWH and being in an HIV cluster by a factor of 3.52 (95% CI 0.11–0.91).

In the full model (Model 7), including both dyadic and network features, the results change considerably. First, concordant STI negative status is associated with a 81% decrease in the odds of LWH or LWH and being in an HIV cluster, significant at the 1% level (95% CI 0.06–0.56). Second, when a participant nominates one more friend or sex partner, the odds of LWH or LWH and being in an HIV cluster decrease by 32% (95% CI 0.59, 0.78). Finally, a unit increase in the constraint score reduced the odds of LWH or LWH and being in an HIV cluster by 90% (95% CI 0.02–0.45). The effect of constraint in model 7 was a different direction than Model 6 and will be discussed later.

We also conducted an ego-level analysis by converting all variables into their average in each ego network to summarize an ego’s social environment and its impact on HIV status and cluster membership. The size and statistical significance of the effects were similar to those in Table 3, but not shown.

## Discussion

This study provides knowledge on how social network structure and dyadic characteristics relate to molecular HIV status and cluster membership among Black and Latino sexual minority men. While prior HIV prevention efforts have largely centered on individual-level behaviors (e.g., condom use, substance use)^34,35^, our findings underscore the critical role of social networks in shaping HIV status and cluster membership.

Consistent with prior research demonstrating the importance of social environments on HIV risk^36,37^, our analysis reveals that network size and cohesion are independently associated with HIV cluster membership. Larger networks and higher constraint (suggestive of less open, more tightly bound networks) were negatively associated with belonging to a HIV transmission cluster. The larger network results align with structural network theories, suggesting that sparse and less cohesive networks may limit repeated exposures within a closed high-risk group, thereby reducing transmission probability^38^. Our results excluding the number of nominees and other dyadic factors suggest that higher constraint predicted molecular clustering. This is consistent with the structural network theories that being embedded in a cohesive network enhances the chance of transmission. However, the full model showed the opposite showing more cohesion as a barrier against the formation of a transmission cluster. As the full model considers the number of nominees and other dyadic factors, this outcome reflects a dynamic discussed by current studies. Within molecular clusters, individuals with more cohesive networks formed a subgroup that acted a firewall. Social cohesion within networks, in this instance, became barriers against viral transmission^39–41^.

Interestingly, racial homophily was associated with increased odds of cluster membership. This supports emerging evidence that homophilous networks, while socially affirming, can perpetuate shared risk environments and facilitate faster viral transmission within racially segregated communities^42,43^. Similarly, concordant STI negative status, a proxy for shared sexual risk, was also linked to a lower likelihood of clustering, perhaps suggesting the existence of concurrent partnerships^44^.

Notably, sociodemographic characteristics such as gender identity and socioeconomic status were not independently associated with HIV status and cluster membership, echoing findings from previous molecular studies that structural inequities may manifest more strongly through their influence on network composition and partner availability than through direct associations with transmission^27,45^.

This study has several limitations. First, the cross-sectional design limits causal inference regarding network characteristics and HIV status and cluster membership. Second, data on social and sexual networks were self-reported and may be subject to recall or social desirability bias. Third, while molecular clustering provides insight into recent transmission, it cannot confirm direct transmission between individuals. Finally, the sample size was modest and limited to one geographic region, which may affect generalizability to other populations or settings. That said, our findings have practical implications for HIV prevention and molecular surveillance. Interventions engaging socially and racially homophilous networks, especially those with low STI concordance, may be effective in interrupting transmission chains. Example interventions could include identifying centrally positioned individuals withing racially homogeneous networks to be HIV and STI prevention and care ambassadors. Moreover, integration of network-based metrics into cluster detection strategies could improve the timeliness and specificity of public health responses^46^. Cluster based network intervention ideas could include extending traditional partner services to social networks and providing PrEP to HIV negative members of individuals in HIV clusters.

Future work should explore the temporal dynamics of these networks and incorporate longitudinal and qualitative data to better understand the mechanisms linking network structure to HIV clustering.

## Conclusion

Our findings suggest that participants are more likely to be in HIV clusters when their networks are more racially homogenous and ethnically heterogeneous. Meanwhile, our sample in Raleigh-Durham reflected that participants who are living with HIV and in HIV clusters nominate fewer people and have less dense networks. Network-informed interventions, coupled with molecular cluster analysis, may offer a more equitable and effective approach to stemming the HIV epidemic among Black/African American or Latin/Hispanic sexual minority men.

## Data Availability

Data are available upon request.

## Appendix

See Table

**Table 4:** Ego race and ethnicity distribution.

	Ethnicity			
Race	Non-hispanic	Hispanic	Refuse to answer	NA
White	4	8	0	1
American Indian	1	1	0	0
Alaskan Native	0	0	0	0
Asian	0	0	1	0
Native Hawaiian	0	0	0	0
Pacific Islander	1	0	0	0
Black/African American	73	6	1	2
Does not want to report	0	0	0	0
Other	1	7	1	0

^4^.
